# An open-framework borophosphate, LiCu_2_BP_2_O_8_(OH)_2_

**DOI:** 10.1107/S1600536809015554

**Published:** 2009-04-30

**Authors:** Juan Zheng, Aiyun Zhang

**Affiliations:** a Department of Physics and Chemistry, Henan Polytechnic University, Jiaozuo 454000, People’s Republic of China

## Abstract

The open-framework alkaline-earth metal borophosphate, lithium dicopper(II) borophosphate dihydroxide, LiCu_2_BP_2_O_8_(OH)_2_, was synthesized hydro­thermally. Its structure may be regarded as a layer formed *via* BO_4_ and PO_4_ tetra­hedra bonding together with distorted CuO_6_ and LiO_6_ octa­hedral units. Each P atom is connected to B, Li and Cu atoms through a bridging O atom. The B atom lies on a crystallographic twofold axis and the Li atom lies on a center of symmetry. The two metal centers are connected to each other by Cu—O—Li bonds.

## Related literature

For chiral structures and potential applications in catalysis of borophosphates with the general formula *AM*(H_2_O)_2_[BP_2_O_8_].*y*H_2_O (*A* = Li, Na, K, NH_4_^+^; *M* = Mg, Mn, Fe, Co, Ni, Cu, Zn, Cd) (*y* = 0.5–1), see: Ewald *et al.* (2007 ▶); Kniep *et al.* (1997 ▶). For related structures, see: Boy & Kniep (2001 ▶); Yang *et al.* (2008 ▶).

## Experimental

### 

#### Crystal data

LiCu_2_BP_2_O_8_(OH)_2_
*M* *_r_* = 368.79
Monoclinic, 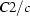

*a* = 15.0974 (19) Å
*b* = 4.7617 (6) Å
*c* = 9.6585 (12) Å
β = 91.0190 (10)°
*V* = 694.23 (15) Å^3^
*Z* = 4
Mo *K*α radiation
μ = 6.64 mm^−1^
*T* = 296 K
0.20 × 0.18 × 0.17 mm

#### Data collection

Bruker APEXII CCD diffractometer
Absorption correction: multi-scan (*SADABS*; Bruker, 2007 ▶) *T*_min_ = 0.350, *T*_max_ = 0.398 (expected range = 0.285–0.324)
4076 measured reflections
925 independent reflections
897 reflections with *I* > 2σ(*I*)
*R*_int_ = 0.032

#### Refinement

*R*[*F* ^2^ > 2σ(*F* ^2^)] = 0.019
*wR*(*F* ^2^) = 0.054
*S* = 1.18
925 reflections
79 parameters
1 restraint
H atoms treated by a mixture of independent and constrained refinement
Δρ_max_ = 0.63 e Å^−3^
Δρ_min_ = −0.53 e Å^−3^

### 

Data collection: *APEX2* (Bruker, 2007 ▶); cell refinement: *SAINT* (Bruker, 2007 ▶); data reduction: *SAINT*; program(s) used to solve structure: *SHELXS97* (Sheldrick, 2008 ▶); program(s) used to refine structure: *SHELXL97* (Sheldrick, 2008 ▶); molecular graphics: *SHELXTL* (Sheldrick, 2008 ▶); software used to prepare material for publication: *SHELXTL*.

## Supplementary Material

Crystal structure: contains datablocks I, global. DOI: 10.1107/S1600536809015554/br2104sup1.cif

Structure factors: contains datablocks I. DOI: 10.1107/S1600536809015554/br2104Isup2.hkl

Additional supplementary materials:  crystallographic information; 3D view; checkCIF report

## Figures and Tables

**Table 1:** Hydrogen-bond geometry (Å, °)

*D*—H⋯*A*	*D*—H	H⋯*A*	*D*⋯*A*	*D*—H⋯*A*
O4—H4⋯O2^i^	0.848 (10)	2.37 (3)	2.9535 (19)	126 (3)
O4—H4⋯O5^ii^	0.848 (10)	2.32 (2)	3.036 (2)	143 (3)

Symmetry codes: (i) 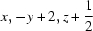; (ii) 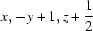.

**Fig. 1.**
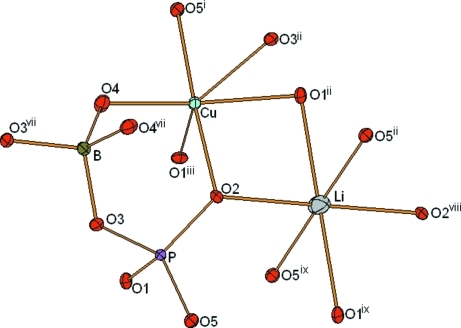
The structure of LiCu2BP2O8(OH)2. Displacement ellipsoids are drawn at 50% the probability level.

**Fig. 2.**
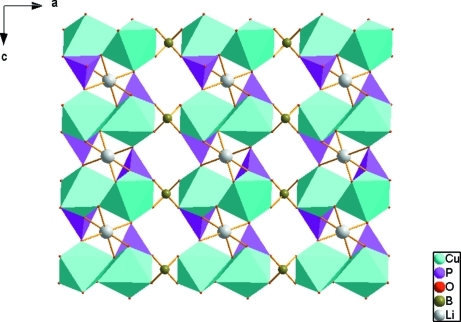
Packing diagram of LiCu2BP2O8(OH)2,viewed along b axis.

## supplementary crystallographic information

### Comment

In the last decade, much attention has been paid to the large family of borophosphates with the general formula *AM*(H_2_O)_2_[BP_2_O_8_].yH_2_O (A=Li, Na, K, NH4^+^; *M*=Mg, Mn, Fe, Co, Ni, Cu, Zn, Cd) (*y *= 0.5–1) due to their chiral structure property and potential applications for catalysts (Kniep *et al.*, 1997; Ewald *et al.*, 2007).

The crystal structure of LiCu_2_BP_2_O_8_(OH)_2 _contains one unique Li atom, two Cu atoms, one boron atom, two phosphor atoms, and eight oxygen atoms and two –OH groups in the asymmetric unit of the framework. The borophosphate units are isolated anions linked by the bonds them form to Cu, Li and H. (Fig.1) Each BO_4_ tetrahedron belongs to the adjacent CuO_6_ octahedra.  The phosphorous atoms are allocated in regular tetrahedral environments with four types of oxygen atoms.  Bond lengths and angles within the anionic partial structure are consistent with related borophosphates (Boy *et al.*, 2001; Yang *et al.* 2008),. Li^+^ is coordinated by the oxygen functions groups of PO_4 _groups.  Cu^2+^ is adjacent to six oxygen atoms, five from PO_4 _groups and one BO_4_ groups, but one of the five PO_4_ links (O3) is also bonded to BO_4_ (Fig.2)

### Experimental

Blue block crystals were synthesized hydrothermally from a mixture of Cu(NO_3_)_2_, Li_2_B_4_O_7_,water and H_3_PO_4_. In a typical synthesis, 0.725 g Cu(NO_3_)_2_ were dissolved in a mixture of 5 mL water, 1.691 g Li_2_B_4_O_7_ and 2 ml (85%) H_3_PO_4 _with constant stirring. Finally,the mixture was kept in a 30 ml Teflon–lined steel autoclave at 443 K for 6days.The autoclave was slowly cooled to room temperature. Blue block crystals of thetitle compound were obtained.

### Refinement

The H atoms of the coordinated water molecule were refined with Uiso(H)=2.4Ueq(O)and distance restraints d(O-H)of 0.86 (1)Å. The highest peak in the difference map is 0.63e/Å, and 0.77Å from O2, and the minimum peak is -0.53e/Å, and 0.70Å from Cu1.

### Crystal data

**Table:**

LiCu_2_BP_2_O_8_(OH)_2_	*F*(000) = 712
*M**_r_* = 368.79	*D*_x_ = 3.528 Mg m^−^^3^
Monoclinic, *C*2/*c*	Mo *K*α radiation, λ = 0.71073 Å
Hall symbol: -C 2yc	Cell parameters from 3236 reflections
*a* = 15.0974 (19) Å	θ = 2.7–29.3°
*b* = 4.7617 (6) Å	µ = 6.64 mm^−^^1^
*c* = 9.6585 (12) Å	*T* = 296 K
β = 91.019 (1)°	Block, blue
*V* = 694.23 (15) Å^3^	0.20 × 0.18 × 0.17 mm
*Z* = 4	

### Data collection

**Table:**

Bruker APEXII CCD diffractometer	925 independent reflections
Radiation source: fine-focus sealed tube	897 reflections with *I* > 2σ(*I*)
graphite	*R*_int_ = 0.032
φ and ω scans	θ_max_ = 29.3°, θ_min_ = 2.7°
Absorption correction: multi-scan (*SADABS*; Bruker, 2007)	*h* = −20→20
*T*_min_ = 0.350, *T*_max_ = 0.398	*k* = −6→6
4076 measured reflections	*l* = −12→12

### Refinement

**Table:**

Refinement on *F*^2^	Secondary atom site location: difference Fourier map
Least-squares matrix: full	Hydrogen site location: inferred from neighbouring sites
*R*[*F*^2^ > 2σ(*F*^2^)] = 0.019	H atoms treated by a mixture of independent and constrained refinement
*wR*(*F*^2^) = 0.054	*w* = 1/[σ^2^(*F*_o_^2^) + (0.0258*P*)^2^ + 1.3109*P*] where *P* = (*F*_o_^2^ + 2*F*_c_^2^)/3
*S* = 1.18	(Δ/σ)_max_ = 0.001
925 reflections	Δρ_max_ = 0.63 e Å^−^^3^
79 parameters	Δρ_min_ = −0.52 e Å^−^^3^
1 restraint	Extinction correction: *SHELXL97* (Sheldrick, 2008), Fc^*^=kFc[1+0.001xFc^2^λ^3^/sin(2θ)]^-1/4^
Primary atom site location: structure-invariant direct methods	Extinction coefficient: 0.0350 (13)

### Special details

**Table:**

Geometry. All e.s.d.'s (except the e.s.d. in the dihedral angle between two l.s. planes) are estimated using the full covariance matrix. The cell e.s.d.'s are taken into account individually in the estimation of e.s.d.'s in distances, angles and torsion angles; correlations between e.s.d.'s in cell parameters are only used when they are defined by crystal symmetry. An approximate (isotropic) treatment of cell e.s.d.'s is used for estimating e.s.d.'s involving l.s. planes.
Refinement. Refinement of F^2^ against ALL reflections. The weighted *R*-factor *wR* and goodness of fit S are based on F^2^, conventional *R*-factors *R* are based on F, with F set to zero for negative F^2^. The threshold expression of F^2^ > 2sigma(F^2^) is used only for calculating *R*-factors(gt) *etc*. and is not relevant to the choice of reflections for refinement. *R*-factors based on F^2^ are statistically about twice as large as those based on F, and R– factors based on ALL data will be even larger.

### Fractional atomic coordinates and isotropic or equivalent isotropic displacement parameters (Å^2^)

**Table:**

	*x*	*y*	*z*	*U*_iso_*/*U*_eq_	
Cu1	0.350210 (15)	1.20604 (5)	0.77155 (2)	0.00731 (13)	
P2	0.35630 (3)	0.67849 (9)	0.58833 (5)	0.00503 (14)	
O3	0.44649 (9)	0.5916 (3)	0.65917 (14)	0.0085 (3)	
O2	0.34451 (9)	0.9972 (3)	0.59584 (13)	0.0080 (3)	
O1	0.28531 (9)	0.5146 (3)	0.66790 (13)	0.0074 (3)	
O5	0.35401 (9)	0.5717 (3)	0.44007 (13)	0.0085 (3)	
O4	0.44543 (9)	0.9550 (3)	0.83773 (13)	0.0101 (3)	
B	0.5000	0.7756 (6)	0.7500	0.0069 (5)	
Li	0.2500	1.2500	0.5000	0.0201 (11)	
H4	0.425 (2)	0.852 (6)	0.901 (2)	0.024*	

### Atomic displacement parameters (Å^2^)

**Table:**

	*U*^11^	*U*^22^	*U*^33^	*U*^12^	*U*^13^	*U*^23^
Cu1	0.01075 (17)	0.00604 (17)	0.00510 (16)	0.00214 (8)	−0.00107 (9)	−0.00075 (7)
P2	0.0066 (2)	0.0042 (2)	0.0042 (2)	−0.00019 (15)	−0.00068 (16)	0.00003 (15)
O3	0.0084 (6)	0.0069 (6)	0.0100 (6)	0.0005 (5)	−0.0034 (5)	−0.0008 (5)
O2	0.0118 (6)	0.0048 (6)	0.0072 (6)	0.0011 (5)	−0.0018 (5)	−0.0006 (5)
O1	0.0082 (6)	0.0068 (6)	0.0073 (6)	0.0005 (5)	0.0014 (4)	0.0022 (5)
O5	0.0130 (7)	0.0076 (6)	0.0049 (6)	0.0003 (5)	−0.0001 (5)	−0.0001 (5)
O4	0.0119 (6)	0.0116 (7)	0.0067 (6)	0.0041 (5)	0.0004 (5)	0.0000 (5)
B	0.0079 (13)	0.0056 (12)	0.0073 (13)	0.000	−0.0006 (10)	0.000
Li	0.021 (3)	0.017 (2)	0.022 (3)	0.006 (2)	−0.012 (2)	−0.006 (2)

### Geometric parameters (Å, °)

**Table:**

Cu1—O5^i^	1.9415 (13)	P2—O1	1.5420 (14)
Cu1—O2	1.9674 (14)	P2—O3	1.5686 (14)
Cu1—O4	1.9676 (14)	B—O4^iv^	1.467 (2)
Cu1—O1^ii^	2.0213 (13)	B—O3^iv^	1.471 (2)
Cu1—O1^iii^	2.3242 (13)	Li—O2^v^	2.0723 (14)
P2—O5	1.5195 (13)	Li—O1^ii^	2.1143 (13)
P2—O2	1.5300 (15)	Li—O5^ii^	2.2758 (14)
			
O5^i^—Cu1—O2	177.21 (6)	P2—O5—Cu1^viii^	127.40 (8)
O5^i^—Cu1—O4	92.77 (6)	P2—O5—Li^vi^	89.42 (6)
O2—Cu1—O4	89.64 (6)	Cu1^viii^—O5—Li^vi^	124.75 (6)
O5^i^—Cu1—O1^ii^	91.48 (6)	B—O4—Cu1	125.51 (9)
O2—Cu1—O1^ii^	85.80 (6)	O4^iv^—B—O4	108.8 (2)
O4—Cu1—O1^ii^	161.34 (6)	O4^iv^—B—O3	108.09 (7)
O5^i^—Cu1—O1^iii^	90.91 (5)	O4—B—O3	112.53 (8)
O2—Cu1—O1^iii^	89.63 (5)	O4^iv^—B—O3^iv^	112.53 (8)
O4—Cu1—O1^iii^	108.74 (6)	O4—B—O3^iv^	108.09 (7)
O1^ii^—Cu1—O1^iii^	89.35 (3)	O3—B—O3^iv^	106.9 (2)
O5—P2—O2	112.09 (8)	O2—Li—O2^v^	180.0
O5—P2—O1	107.21 (8)	O2—Li—O1^ii^	80.86 (5)
O2—P2—O1	113.33 (8)	O2^v^—Li—O1^ii^	99.14 (5)
O5—P2—O3	109.13 (8)	O2—Li—O1^ix^	99.14 (5)
O2—P2—O3	109.99 (8)	O2^v^—Li—O1^ix^	80.86 (5)
O1—P2—O3	104.75 (7)	O1^ii^—Li—O1^ix^	180.0
B—O3—P2	124.48 (13)	O2—Li—O5^ii^	91.82 (5)
P2—O2—Cu1	122.61 (8)	O2^v^—Li—O5^ii^	88.18 (5)
P2—O2—Li	129.39 (8)	O1^ii^—Li—O5^ii^	68.18 (5)
Cu1—O2—Li	96.37 (6)	O1^ix^—Li—O5^ii^	111.82 (5)
P2—O1—Cu1^vi^	106.24 (7)	O2—Li—O5^ix^	88.18 (5)
P2—O1—Li^vi^	95.01 (6)	O2^v^—Li—O5^ix^	91.82 (5)
Cu1^vi^—O1—Li^vi^	93.45 (6)	O1^ii^—Li—O5^ix^	111.82 (5)
P2—O1—Cu1^vii^	123.34 (8)	O1^ix^—Li—O5^ix^	68.18 (5)
Cu1^vi^—O1—Cu1^vii^	125.56 (6)	O5^ii^—Li—O5^ix^	180.0
Li^vi^—O1—Cu1^vii^	102.45 (5)		

Symmetry codes: (i) *x*, −*y*+2, *z*+1/2; (ii) *x*, *y*+1, *z*; (iii) −*x*+1/2, *y*+1/2, −*z*+3/2; (iv) −*x*+1, *y*, −*z*+3/2; (v) −*x*+1/2, −*y*+5/2, −*z*+1; (vi) *x*, *y*−1, *z*; (vii) −*x*+1/2, *y*−1/2, −*z*+3/2; (viii) *x*, −*y*+2, *z*−1/2; (ix) −*x*+1/2, −*y*+3/2, −*z*+1.

### Hydrogen-bond geometry (Å, °)

**Table:**

*D*—H···*A*	*D*—H	H···*A*	*D*···*A*	*D*—H···*A*
O4—H4···O2^i^	0.85 (1)	2.37 (3)	2.9535 (19)	126 (3)
O4—H4···O5^x^	0.85 (1)	2.32 (2)	3.036 (2)	143 (3)

Symmetry codes: (i) *x*, −*y*+2, *z*+1/2; (x) *x*, −*y*+1, *z*+1/2.
